# The impact of frailty syndrome on humoral response to SARS-CoV-2 mRNA vaccines in older kidney transplant recipients

**DOI:** 10.1007/s11255-023-03557-6

**Published:** 2023-04-07

**Authors:** Michal Schmalz, Hana Vankova, Silvie Rajnochova-Bloudickova, Petra Hruba, Martina Fialova, Jiri Gurka, Maria Magicova, Ilja Striz, Ivan Zahradka, Ondrej Viklicky

**Affiliations:** 1 Department of Nephrology, Institute for Clinical and Experimental Medicine, Prague, Czech Republic; 2 Department of Immunology, Institute for Clinical and Experimental Medicine, Prague, Czech Republic; 3 Information Technology Department, Institute for Clinical and Experimental Medicine, Prague, Czech Republic; 4 Transplantation Laboratory, Institute for Clinical and Experimental Medicine, Prague, Czech Republic; 5 The Third Faculty of Medicine, Charles University, Prague, Czech Republic

**Keywords:** Frailty, Kidney transplantation, SARS-CoV-2 infection, Vaccination

## Abstract

**Purpose:**

Advanced age is associated with an impaired humoral immune response to SARS-CoV-2 mRNA vaccination in kidney transplant recipients (KTR). The mechanisms are, however, poorly understood. Frailty syndrome assessment may determine the most vulnerable population.

**Methods:**

This study is a secondary analysis of a prospective study (NCT04832841) regarding seroconversion after BNT162b2 vaccination, including 101 SARS-CoV-2 naïve KTR 70 years and older. The Fried frailty components were evaluated, and antibodies against S1 and S2 subunits of SARS-CoV-2 were examined > 14 days after the second dose of BNT162b2 vaccine.

**Results:**

Seroconversion was observed in 33 KTR. Male gender, eGFR, MMF-free immunosuppression, and a lower frailty score were associated with higher seroconversion rates in univariable regression. Concerning frailty components, physical inactivity had the most negative effect on seroconversion (OR = 0.36, 95% CI 0.14–0.95, *p* = 0.039). In a multivariable regression adjusted for eGFR, MMF-free immunosuppression, time from transplant and gender, pre-frail (OR = 0.27, 95% CI 0.07–1.00, *p* = 0.050), and frail status (OR = 0.14, 95% CI 0.03–0.73, *p* = 0.019) were associated with an increased risk of unresponsiveness to SARS-CoV-2 vaccines.

**Conclusion:**

Frailty was associated with an impaired humoral response to SARS-CoV-2 mRNA vaccination in older SARS-CoV-2 naïve KTR*.*

**Trail registration:**

This study is registered under the identifier NCT04832841 on ClinicalTrials.gov.

**Supplementary Information:**

The online version contains supplementary material available at 10.1007/s11255-023-03557-6.

## Introduction

Kidney transplant recipients (KTR) are considered at high risk for COVID-19 infection-associated morbidity and mortality [1]. Vaccination of this vulnerable population has, therefore, been recommended. However, recent studies have revealed a significantly impaired humoral response to mRNA vaccines in KTR compared to the general population [2]. The use of mycophenolate mofetil, depletive induction therapy, and costimulation blockade, as well as older age were found to be among the main risk factors of limited humoral response to SARS-CoV-2 mRNA vaccines [3–5] .

With improvements in KTR survival rates [6], elderly patients constitute a significant part of the KTR population. Elderly patients are at higher risk for infectious complications, which is frequently further aggravated by frailty syndrome [7]. Frailty syndrome, characterized by decreased physiologic reserve and resistance to stressors, further increases COVID-19-associated morbidity and mortality [1, 8].

Some previous studies have considered frailty a risk factor for decrease in flu vaccine effectiveness in the general population [9]. However, their findings are conflicting [10, 11]. Currently available data concerning the effect of frailty on seroconversion rates after SARS-CoV-2 vaccination in the general population are limited, and to the best of our knowledge, no study has yet pointed toward an association between frailty and impaired seroconversion in KTR [12]. We, therefore, aimed to assess the humoral response following vaccination with the mRNA SARS-CoV-2 vaccine (BNT162b2) in frail and non-frail elderly kidney transplant recipients.

## Methods

### Study design

**Fig. 1:**
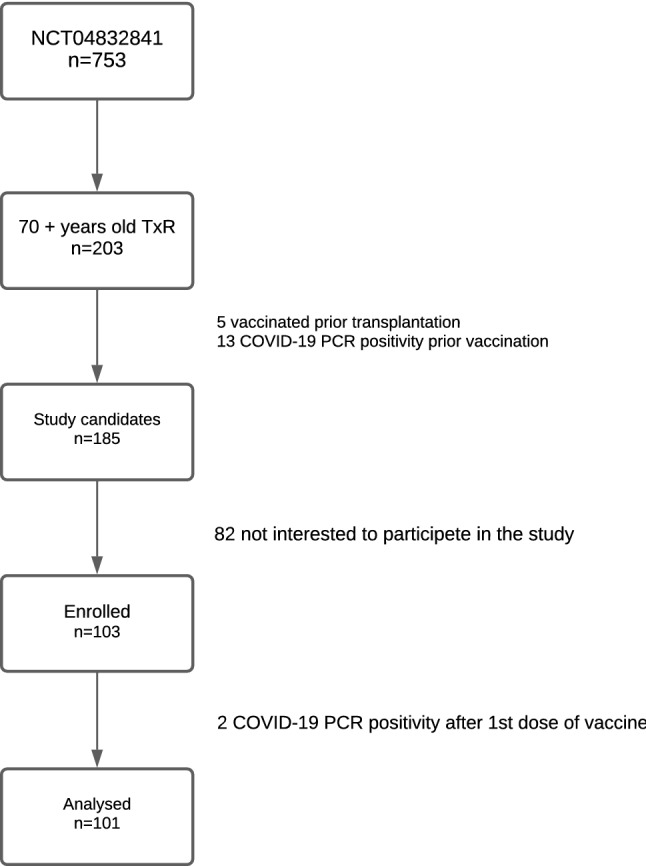
Study flowchart. A total of 203 kidney transplant recipients (KTR) from the original study (NCT04832841) were 70 and more years old. Five KTR were vaccinated while on the waiting list prior to transplantation, thirteen were COVID-19 positive prior to vaccination and eighty-two KTR were enrolled in the original study but were not interested to participate in the frailty assessment. A total of 103 KTR were enrolled in this study and of them 2 experienced COVID-19 PCR positivity after the 1st vaccine dose. Thus, a total of 101 KTR were included in the final analysis

This is a secondary analysis of a prospective single-center study (NCT04832841) performed in 2021 (April–June) [5]. Our objective is to assess the seroprevalence of anti SARS-CoV-2 S1/S2 IgG assessment following SARS-CoV-2 mRNA vaccination in KTR. Frailty assessment was offered to KTR 70 years and older who were SARS-CoV-2 naïve and had received two doses of mRNA BNT162b2 vaccine. Antibody testing was done at least 14 days after the second dose of BNT162b2 vaccine. Other inclusion and exclusion criteria and a description of antibody testing have already been reported [5, 13]. Finally, 103 KTRs were enrolled in the study (Fig. 1). Two KTR were excluded due to a positive SARS-CoV-2 PCR test following the first vaccine dose and eighty-two KTR declined frailty assessment.

### Frailty assessment

Frailty was evaluated according to Fried frailty criteria and on the same day, blood samples for antibody testing were collected. Frailty was determined based on five components: weight loss (self-reported unintentional weight loss of more than 10 lbs in the past year); weakness (grip strength below an established cutoff based on gender and BMI); exhaustion (self-reported); physical inactivity (kcals/week below an established cutoff); slower walking speed (walking time of 15 feet below an established cutoff by gender and height) [8]. Each of the five components was scored 0 or 1 depending on the absence or the presence of the component, respectively. The cumulative Fried frailty score was calculated as the sum of the component scores (range 0–5). Non-frail status was defined as a score of 0, pre-frail status was defined as a score of 1–2, and frail status was defined as a score of 3 and higher [8].

### Statistical analysis

Continuous variables are expressed as medians (min, max) and categorical variables are expressed as *n* and a percentage of the total and compared by Pearson Chi-square test. Univariable and multivariable binary regression analyses were used to predict the responsiveness to SARS-CoV-2 vaccination. The multivariable binary logistic regression model was based on the multivariable model of the primary study and included a variable for frailty as well as variables which were previously found to be independently associated with vaccine immune response. The independent variables associated with vaccine immune response in the primary study were age, gender, time from transplant to vaccination, eGFR, mycophenolate use, and depleting therapy in the last year [5]. However, age was not included in the current final model, as all the KTR were elderly within few years of each other. Furthermore, no elderly KTR received depleting therapy in the last year, and thus this variable could also not be included. Aside from mycophenolate use, no other immunosuppression variable was entered into the model, as none were independently associated with vaccine response in the original study. Sensitivity analyses of multivariable binary logistic regression model was calculated using bootstrap resampling. Additional sensitivity analysis was performed where all statisticaly significant variables in univariable regression were also included in the multivariable model. A *p *value of less than 0.05 was considered statistically significant. There were no missing data of variables of interest. Statistical analysis was performed using IBM SPSS Statistics, Version 24 (International Business Machines Corp.) and R-studio version 4.0.3. (2020-10-10).

## Results

A total of 101 elderly KTR naïve to COVID-19 were included in the analysis. Males and females were represented equally. The median age was 73 years, most patients had undergone the first transplantation, and the median follow-up from transplantation was 6.2 years. Details on demography and immunosuppression are provided in Table 1. In the entire cohort, seroconversion after two doses of mRNA BNT162b2 vaccine was observed in 33% KTRs who were older than 70 years of age.

**Table 1:** General characteristics of the study cohort

	Study cohort, *n* = 101
Male sex—no. (%)	51 (50%)
Age in years—median (min–max)	73 (70–82)
Number of transplant (1st/2nd/3rd)—no. (%)	95(94%)/5(5%)/1(1%)
BMI—median (min–max)	28.4 (16–41)
Living donor—no. (%)	6 (6%)
Time from transplant in years—median (min–max)	6.2 (0.5–26.6)
Dialysis vintage in years—median (min–max)	2 (0.6–8)
Diabetes mellitus—no. (%)	42 (42%)
eGFR in ml/min/1.73m^2^—median (min–max)^a^	46.2 (10.2–94.8)
CNI-based IS—no. (%)	85 (84%)
Tacrolimus based IS—no. (%)	71 (70%)
CyA based IS—no. (%)	14 (14%)
MMF / MPA—no. (%)	75 (74%)
mTORi—no. (%)	13 (13%)
Depletive induction—no. (%)	37 (37%)
Antirejection therapy—no. (%)	19 (19%)
Time from 2nd dose to Ab testing in days—median (min–max)	66 (19–114)

*BMI* body mass index, *CNI-based* calcineurin inhibitor-based, *CyA* cyclosporin A, *MMF* mycophenolate mofetil, *MPA* mycophenolic acid, *mTORi* mammalian target of rapamycin inhibitor

^a^Serum creatinine measurements were done on the day of antibody testing

Twenty-seven (27%) patients were classified as frail and forty-eight (48%) patients as pre-frail. General characteristics of patients divided by frailty status are provided in the Supplementary Table 1. Exhaustion (45%) and physical inactivity (36%) were the most frequently observed Fried frailty components. Weakness, slow walking speed, and weight loss were observed in 29%, 24%, and 12%, respectively. Seroconversion after vaccination was observed in 12 out of 27 (44%) non-frail, 15 out of 48 (31%) pre-frail, and 6 out of 26 (23%) frail KTR, respectively. Interestingly, there were 36 (36%) KTR who were physically inactive, and 29 of them (80%) did not mount antibodies after vaccination (*p* = 0.046) (Table 2).

**Table 2:** Comparison of the frailty characteristic of responders and non-responders to SARS-CoV-2 mRNA vaccination

	Non-responders (*n* = 68)	Responders (*n* = 33)	*p* value^a^
*Frailty phenotype*			0.243
Non-frail status—no. (%)	15 (22%)	12(44%)	
Pre-frail status—no. (%)	33 (49%)	15 (45%)	
Frail status—no. (%)	20 (29%)	6 (18%)	
*Fried frailty components*			
1 Physical inactivity—no. (%)	29 (43%)	7 (19%)	0.046
2 Weakness—no. (%)	21 (31%)	8 (24%)	0.640
3 Slowed walking speed—no. (%)	16 (24%)	8 (24%)	1
4 Exhaustion—no. (%)	34 (50%)	11 (33%)	0.138
5 Weight loss—no. (%)	9 (13%)	3 (9%)	0.747
Fried cumulative score (0–5)—mean (standard deviation)	1.6 (1.33)	1.12 (1.29)	0.051

*mRNA* messenger ribonucleic acid, *SARS-CoV-2* severe acute respiratory syndrome coronavirus 2

^a^*p* values for group comparison based on the Mann–Whitney *U* test for continuous variables, Pearson’s Chi-squared test for categorical variables, *p* < 0.05 for significance

Seroconversion was further used as the dependent variable for univariable and multivariable binary logistic regression. The variables associated with seroconversion in univariable regression were eGFR (OR = 1.03, 95% CI 1.01–1.06, *p* = 0.001), MMF-free immunosuppression (IS) regimen (OR = 4.32, 95% CI 1.69–11.07, *p* = 0.002), CNI-based IS (OR = 0.22, 95% CI 0.07–068, *p* = 0.009), and mTOR inhibitor IS (OR = 4.03, CI 1.21–13.52, *p* = 0.024). Male gender (OR = 2.09, 95% CI 0.89–4.9, *p* = 0.091), depletive induction IS (OR = 0.43, CI 0.17–1.09, *p* = 0.076), and Fried frailty cumulative score (OR = 0.74, 95% CI 0.52–1.05, *p* = 0.09) did not reach statistical significance. Age did not affect seroconversion in our cohort (OR = 1.07, 95% CI 0.95–1.2, *p* = 0.267) (Table 3).

**Table 3:** Determinants of seroconversion in elderly SARS-CoV-2 naïve KTR, univariable analysis

Univariable analysis^a^			
Variable	OR	95% CI	*p* value
Gender (male)	2.09	0.89–4.9	0.091
Age (years)	1.07	0.95–1.2	0.267
Retransplantation	2.17	0.41–11.37	0.361
BMI	1.01	0.94–1.09	0.741
Living donor	1.03	0.18–5.95	0.972
Time after transplant (years)	1.04	0.99–1.01	0.118
Dialysis vintage before transplant (years)	0.82	0.62–1.08	0.152
Diabetes mellitus	0.68	0.29–1.61	0.380
eGFR (ml/min/1.73 m^2^)	1.03	1.01–1.06	0.001
CNI-based IS	0.22	0.07–0.68	0.009
Tacrolimus based IS	0.34	0.14–0.83	0.018
CyA based IS	1.17	0.36–3.8	0.794
MMF-free immunosuppression	4.32	1.69–11.07	0.002
mTORi	4.03	1.21–13.52	0.024
Depletive induction	0.43	0.17–1.09	0.076
Antirejection therapy	1.26	0.44–3.56	0.668
Time from second vaccine dose to antibody testing (days)	1.01	0.99–1.02	0.500
Fried Frailty cumulative score^b^	0.74	0.52–1.05	0.091
Non-frail status	Reference category		
Pre-frail status	0.57	0.22–1.51	0.255
Frail status	0.38	0.11–1.23	0.105

*CI* confidence interval, *MMF* mycophenolate mofetil, *mRNA* messenger ribonucleic acid, *SARS-CoV-2* severe acute respiratory syndrome coronavirus 2, *eGFR* estimated glomerular filtration rate, *OR* odds ratio

^a^Univariable associations were calculated by binary logistic regression, results are expressed with odds ratios and their 95% confidence intervals, *p* < 0.05 for significance

^b^Fried Frailty cumulative score inputted as a continuous variable

**Fig. 2:**
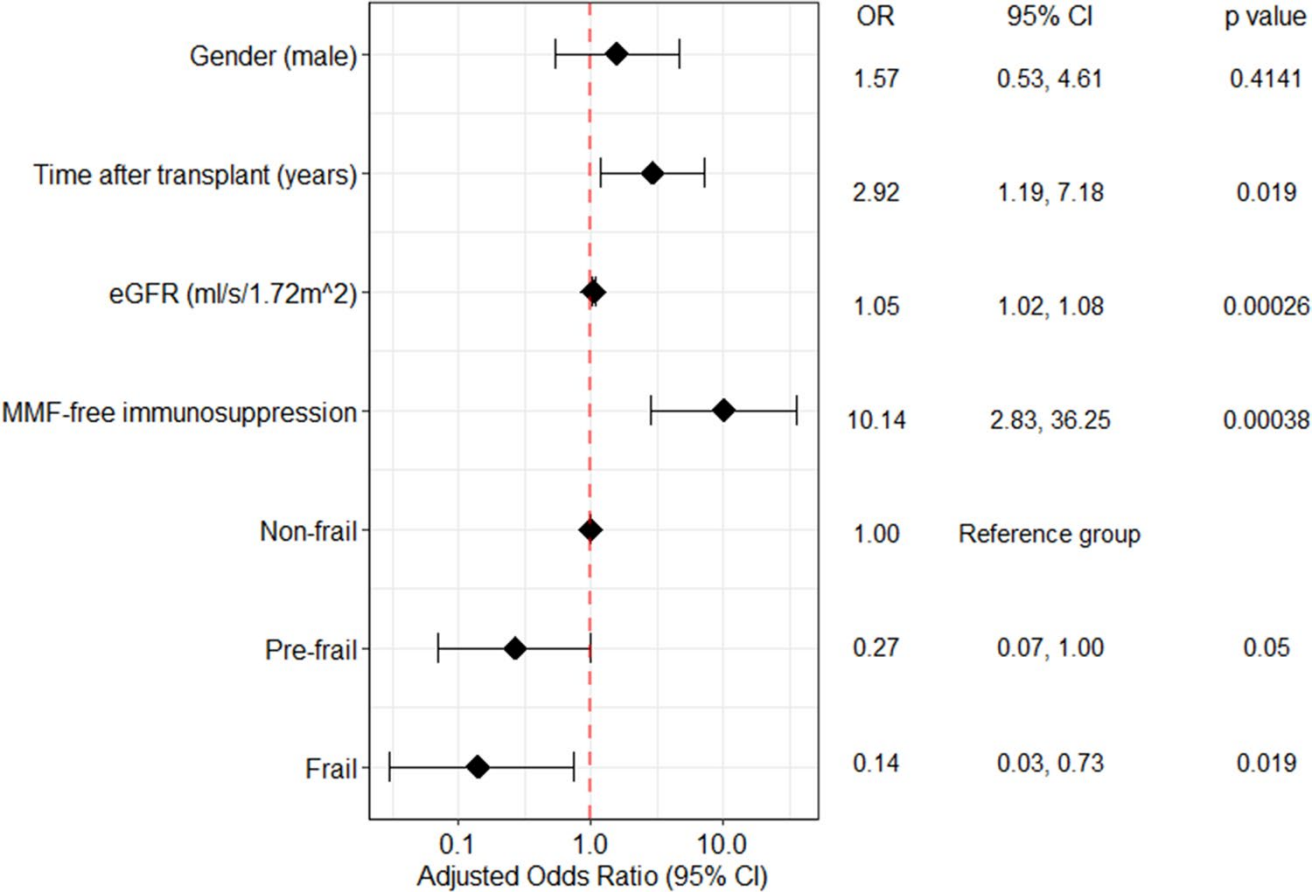
Determinants of seroconversion after two-dose mRNA vaccination in elderly kidney transplant recipients. Results displayed as a Forest plot (log scale) with adjusted odds ratios and their 95% confidence intervals

Next, a multivariable binary logistic regression model was calculated. Pre-frail (OR = 0.27, 95% CI 0.07–1.00, *p* = 0.050) and frail (OR = 0.14, 95% CI 0.03–0.73, *p* = 0.019) status were associated with an increased risk of unresponsiveness to SARS-CoV-2 vaccination. The model was adjusted for eGFR (OR = 1.05, 95% CI 1.02–1.08, *p* < 0.001), MMF-free immunosuppression (OR = 10.14, 95% CI 2.83–36.25, *p* < 0.001), longer time from transplant (OR = 2.92, 95% CI 1.19–7.18, *p* = 0.019), and gender (Fig. 2). The stability of confidence intervals of all variables in the multivariable binary logistic regression model was checked internally using bootstrap resampling (*n* = 1000; Supplementary Table 2). Furthermore, an additional sensitivity analysis was performed where all statisticaly significant variables in univariable regression were also included in the multivariable model. This approach did not alter the study results (Supplementary Table 3).

Furthermore, Fried frailty components were separately analyzed in univariable regression (Table 4). The most significant negative impact on seroconversion was observed for physical inactivity (OR 0.36, 95% CI 0.14–0.95, *p* = 0.039). Weakness (hand grip strength) alone did not affect seroconversion; however, better seroconversion was observed in KTR with a higher absolute value of maximal hand grip strength (OR = 1.05, CI 95% 1.01–1.11, *p* = 0.045).

**Table 4:** Univariable associations of individual frailty components with seroconversion rate

Univariable analysis^a^			
Variable	OR	95% CI	*p* value
Physical inactivity	0.36	0.14–0.95	0.039
Weight loss	0.68	0.17–2.69	0.581
Weakness	0.72	0.28–1.85	0.404
Exhaustion	0.50	0.21–1.19	0.117
Slow walking speed	1.04	0.39–2.75	0.937

*CI* confidence interval, *mRNA* messenger ribonucleic acid, *SARS-CoV-2* severe acute respiratory syndrome coronavirus 2, *OR* odds ratio

^a^Univariable associations were calculated by binary logistic regression, results are expressed with odds ratios and their 95% confidence intervals, *p* < 0.05 for significance

## Discussion

In this study, we aimed to evaluate the association between frailty and an impaired humoral response to SARS-CoV-2 mRNA vaccination in elderly virus-naïve KTR. We observed a better seroconversion rate in the non-frail group, compared to the pre-frail and frail KTR groups. Furthermore, of the Fried frailty components, physical inactivity had the most negative impact on seroconversion.

Frailty was assessed using Fried frailty criteria, which were previously validated by McAdams-DeMarco in KTR [14]. The prevalence of frailty syndrome was studied in kidney transplant candidates, but less is known about the long-term frailty prevalence in KTR. The group of McAdams-DeMarco observed that 23.7% kidney transplant candidates 65–75 years old and 22.7% transplant candidates older than 75 years old are frail at the time of transplant [15]. Although frailty might be reversible after transplant, it’s incidence rises later in the follow-up period after transplantation [16]. These results support our observation that every fourth elderly KTR was assessed as frail.

Among Fried frailty components, physical inactivity and exhaustion were most frequently observed in our cohort. The group of McAdams-DeMarco described poor grip strength and low physical activity as the most frequent frailty components in KTR at time of transplant, exhaustion was present in 25% of patients [15]. Since the majority of our patients described deconditioning and exhaustion during the pandemic lockdown, we hypothesize that prolonged social isolation due to the COVID-19 pandemic might have a negative impact on physical activity in this vulnerable population [17].

In our study, low physical activity showed the best correlation with humoral response among all the Fried frailty components. This observation is in line with previous reports that studied humoral responses to the flu vaccination and suggested better humoral responses among more physically active older adults [18] and among healthy adults that exercised prior to vaccination [19].

The seroconversion rate in our KTR cohort was only 33% as opposed to the 45.8% observed in the whole cohort in the primary study [5]. While age had a significant impact on seroconversion in the primary study (adults 18 +), we have not found any link between age and seroconversion rates in SARS-CoV-2 naïve KTR older than 70 years of age. The impact of age on vaccination response is poorly understood; however, lower antibody production and T-cell response after the first dose of BNT162b2 vaccine in 80 + years old individuals was previously reported [20]. While number of studies indicated that seroconversion rate in older KTR after SARS-COV-2 vaccination was impaired, our study suggests that the impact of age is no longer relevant among KTR older than 70 years [2, 4, 5].

Similarly to previous studies, we identified an association between MMF therapy and an impaired humoral response to vaccination in frail KTR [21]. Therefore, when analyzing frailty effects on seroconversion, the adjustment for immunosuppression was necessary. Interestingly, all four KTR, who were not frail and did not receive MMF, mounted antibodies after SARS-COV-2 mRNA vaccination. Conversely, seroconversion rate of just 17% was observed in frail KTR treated with MMF. The effect of frailty on the response to flu vaccination was studied in the general population with inconclusive results [11, 22], and data regarding the response to SARS-CoV-2 vaccines in frail subjects are scarce. Ríos et al. [12] showed no effect of frailty on post-vaccination antibody levels among nursing homes residents. However, a major limitation of said study is that majority of the subjects had previously experienced COVID-19 infection and were not virus naïve, which, in turn, biased the observation regarding vaccine-induced antibody production. To the best of our knowledge, our study is the first to assess the effect of frailty on mRNA vaccination response in solid organ recipients.

Based on the findings of our study, we suggest that geriatric assessment might be a useful tool to identify the most vulnerable KTRs at risk of insufficient vaccine response. These patients might, therefore, benefit from interventions such as application of additional vaccine booster doses or passive immunization.

The limitation of our study is the lack of validation cohort and the lack of data concerning the cellular response, as the measurement was not performed.

In conclusion, our study shows a significantly impaired humoral response to SARS-CoV-2 mRNA vaccination in frail elderly KTR who were naïve to SARS-CoV-2.

### Supplementary Information

Below is the link to the electronic supplementary material.

Supplementary file1 (DOCX 22 KB)

Supplementary file2 (DOCX 35 KB)

## Data Availability

The data that support the findings of this study are available from the corresponding author upon request.

## Abbreviations

AU: Arbitrary units
BMI: Body mass index
CNI: Calcineurin inhibitor
COVID-19: Coronavirus disease 2019
eGFR: Estimated glomerular filtration rate
IgG: Immunoglobulin G
IS: Immunosuppression
IU: International units
KTR: Kidney transplant recipients
PCR: Polymerase chain reaction
MMF: Mycophenolate mofetil
mRNA: Messenger ribonucleic acid
SARS-CoV-2: Severe acute respiratory syndrome coronavirus 2
SPSS: Statistical package for the social sciences
