# Supplementary material for: The impact of frailty syndrome on humoral response to SARS-CoV-2 mRNA vaccines in older kidney transplant recipients
Source: Int Urol Nephrol. 2023 Apr 7;55(11):2959–65. doi: 10.1007/s11255-023-03557-6 (PMC10081301; doi:10.1007/s11255-023-03557-6)
Supplement: Supplementary file 1 — Supplementary file1 (DOCX 22 KB) [file 11255_2023_3557_MOESM1_ESM.docx]

**The Impact of frailty syndrome on humoral response to SARS-Cov-2 mRNA vaccines in older kidney transplant recipients**

**International Urology and Nephrology**

Schmalz, Michal, Vankova Hana, Rajnochova-Bloudickova, Silvie, Hruba, Petra, Fialova, Martina,, Gurka, Jiri, , Magicova, Maria, Striz, Ilja , Zahradka, Ivan, Ondrej Viklicky

Institute for Clinical and Experimental Medicine, Prague, Czech Republic ondrej.viklicky@ikem.cz

| **Frailty category** | **Non-frail (n=27)** | **Pre-frail (n=48)** | **Frail (n=26)** |
| --- | --- | --- | --- |
| Age in years – median (min – max) | 71 (70 – 82) | 73 (70 – 82) | 74 (70 – 82) |
| Male sex – no. (%) | 16 (59%) | 27 (56%) | 9 (34%) |
| Time from transplant in years – median (min – max) | 2.5 (0.5 – 27.9) | 6.85 (0,72 –28,5) | 9.5 (0.6 – 27.3) |
| eGFR in ml/min/1,73m^2^ – median (min – max) ^a^ | 48.6 (19.8 – 89.4) | 43.8 (10.2 – 94.8) | 43.2 (13.8 – 90.6) |
| MMF / MPA – no. (%) | 23 (85%) | 33 (68%) | 19 (73%) |

**Supplementary Table 1.** General characteristic divided by frailty category

Abbreviations: MMF, mycophenolate mofetil; MPA, mycophenolic acid;

^a^ Serum creatinine measurements were done on the day of antibody testing.

**Supplementary Table 2.** Results of multivariable regression model and bootstrap sensitivity analyses

|  | **Multivariable regression^a^** | | | **Bootstrap^b^** | |
| --- | --- | --- | --- | --- | --- |
|  | **OR** | **95%Cl** | **P value** | **95%Cl** | **P value** |
| Gender | 1.57 | 0.53–4.61 | 0.414 | -0.770–1.800 | 0.417 |
| Time after transplant (years) | 2.92 | 1.19–7.18 | 0.019 | 0.025–0.186 | 0.005 |
| eGFR(ml/s/1.72m2) | 1.05 | 1.02–1.08 | <0.001 | 0.024–0.092 | 0.001 |
| MMF free immunosuppression | 10.14 | 2.83–36.25 | <0.001 | 1.272–4.329 | 0.001 |
| Non-Frail | Reference group | | | Reference group | |
| Pre-frail | 0.27 | 0.07–1.00 | 0.050 | -3.271–0.023 | 0.060 |
| Frail | 0.14 | 0.03–0.73 | 0.019 | -4.455–-0.306 | 0.015 |

Abbreviations: MMF, mycophenolate mofetil

^a^ Multivariable regression associations were calculated by binary logistic regression, results are expressed with odds ratios and their 95% confidence intervals, p < 0.05 for signifikance.

**^b^** Bootstrap resampling (n=1000) of all variables in the multivariable binary logistic, results are expressed in 95% confidence intervals, p < 0.05 for signifikance.

**Supplementary Table 3.** Results of multivariable regression model based on results of univariable analyses

|  | **Multivariable regression^a^** | | |
| --- | --- | --- | --- |
|  | **OR** | **95%Cl** | **P value** |
| Gender | 1.63 | 0.53–4.98 | 0.394 |
| Time after transplant (years) | 1.07 | 0.98-1.16 | 0.138 |
| eGFR (ml/s/1.73 m^2^) | 1.05 | 1.02-1.08 | 0.001 |
| MMF-free immunosuppression | 13.42 | 3.21-56.11 | <0.001 |
| mTOR inhibitor | 0.73 | 0.09-6.20 | 0.775 |
| CNI-based immunosuppression | 0.14 | 0.18-1.08 | 0.060 |
| Non-Frail | Reference group | | |
| Pre-frail | 0.24 | 0.06–0.93 | 0.039 |
| Frail | 0.11 | 0.02–0.62 | 0.012 |

Abbreviations: CNI, calcineurin inhibitor; MMF, mycophenolate mofetil

^a^ Multivariable regression associations were calculated by binary logistic regression, results are expressed with odds ratios and their 95% confidence intervals, p < 0.05 for signifikance.
